# Snakes elicit earlier, and monkey faces, later, gamma oscillations in macaque pulvinar neurons

**DOI:** 10.1038/srep20595

**Published:** 2016-02-08

**Authors:** Quan Van Le, Lynne A. Isbell, Jumpei Matsumoto, Van Quang Le, Hiroshi Nishimaru, Etsuro Hori, Rafael S. Maior, Carlos Tomaz, Taketoshi Ono, Hisao Nishijo

**Affiliations:** 1 System Emotional Science, Graduate School of Medicine and Pharmaceutical Sciences, University of Toyama, Sugitani 2630, Toyama, 930-0194, Japan; 2 Department of Anthropology, University of California, Davis, CA 95616, USA; 3 Primate Center and Laboratory of Neurosciences and Behavior, Department of Physiological Sciences, Institute of Biology, University of Brasília, CEP 70910-900, Brasilia, DF, Brazil; 4 Vietnam Military Medical University, Ha Noi, Vietnam; 5 Karolinska Institute, Department of Clinical Neuroscience, Psychiatry Section, Karolinska Hospital, S-17176 Stockholm, Sweden; 6 University CEUMA, Neuroscience Research Coordenation, Campus Renascença, CEP 65.075-120 São Luis, MA, Brazil

## Abstract

Gamma oscillations (30–80 Hz) have been suggested to be involved in feedforward visual information processing, and might play an important role in detecting snakes as predators of primates. In the present study, we analyzed gamma oscillations of pulvinar neurons in the monkeys during a delayed non-matching to sample task, in which monkeys were required to discriminate 4 categories of visual stimuli (snakes, monkey faces, monkey hands and simple geometrical patterns). Gamma oscillations of pulvinar neuronal activity were analyzed in three phases around the stimulus onset (Pre-stimulus: 500 ms before stimulus onset; Early: 0–200 ms after stimulus onset; and Late: 300–500 ms after stimulus onset). The results showed significant increases in mean strength of gamma oscillations in the Early phase for snakes and the Late phase for monkey faces, but no significant differences in ratios and frequencies of gamma oscillations among the 3 phases. The different periods of stronger gamma oscillations provide neurophysiological evidence that is consistent with other studies indicating that primates can detect snakes very rapidly and also cue in to faces for information. Our results are suggestive of different roles of gamma oscillations in the pulvinar: feedforward processing for images of snakes and cortico-pulvinar-cortical integration for images of faces.

Animals must be able to navigate the biotic world in order to survive and reproduce successfully. This includes avoiding predators and dealing with conspecifics. The main predators of primates are mammalian carnivores, raptors, and snakes^1^. A combination of neuroscientific, paleontological, molecular, and biogeographic evidence has been used to propose that snakes in particular were largely responsible for the origin of primates and the later appearance of anthropoid primates via selection on the visual system for more rapid and reliable detection^2^,^3^. Despite the great expansion of the primate visual sense compared to other mammals, however, snakes are often highly camouflaged and can be extremely difficult to see, even for primates^4^, and primates, including humans, are still at risk of death from both constricting and venomous snakes today^5^,^6^,^7^. Extensive research has now shown that snakes can elicit reliable behavioral responses from primates, including rapid detection, focused attention, and avoidance^8^,^9^,^10^,^11^,^12^,^13^,^14^,^15^.

Interacting with conspecifics is especially challenging for animals that routinely live together in social groups because social interactions occur frequently between familiar individuals over long periods of time. Most catarrhine primates, including macaques (*Macaca* spp.), live in such groups and have facial musculature that allows a wide range of facial expressions revealing emotion or intention, from threats to appeasement^16^,^17^. Moreover, in catarrhines, facial nucleus volume as an estimate of facial motor control is correlated with the volume of V1^18^. Both the ability to express intentions clearly and the ability of others to process those expressions correctly should also be advantageous to survival and reproduction in social animals.

Responses to faces, facial expressions reflecting threat or danger, and snakes have been associated with the subcortical visual system involving the superior colliculus (SC) and pulvinar, which provides fast and coarse processing of such visual stimuli^10^,^16^,^19^,^20^,^21^,^22^,^23^,^24^,^25^. The pulvinar has uniquely evolved in primates^26^, suggesting that there has been strong selection on primates for rapid visual detection of threatening stimuli. Previous neurophysiological studies reported that neurons in the pulvinar of Japanese macaques (*M. fuscata*) responded even faster and more strongly to snakes than to monkey faces^23^, and more strongly snakes in striking posture than in non-striking posture^27^. These findings suggest that snakes, which can bite extremely quickly, have provided a source of selection for particularly rapid visual responses that can be distinguished from other threats that may be informative but not as time-sensitive.

It is still not clear, however, how snake-induced neuronal firing captures attention so rapidly. Recent studies suggest that gamma oscillation might facilitate perceptual processing in the cortical visual system by feedforward processes^28^,^29^,^30^. The pulvinar plays an important role in regulating cortico-cortical information transmission through the modulation of inter-areal synchrony during cognitive tasks^31^, and a critical role in attentional selection and in regulating information transmission across the visual cortex^32^. When a stimulus is visually attended to, rhythmic gamma band oscillation is induced to establish a communication link among multiple brain areas^33^. Furthermore, there is some evidence that gamma band activity (oscillation) is correlated with a decrease in reaction time^34^,^35^. These findings suggest that snakes might elicit gamma oscillations in the pulvinar. In the present study, we analyzed gamma oscillations of the neuronal responses in the monkey pulvinar to images of snakes, and compared them with monkey faces and, as controls, monkey hands and simple geometrical patterns to complement an earlier study that documented preferential neuronal responses in the pulvinar to snakes^23^. We predicted that gamma oscillations would occur earlier in response to images of snakes than for other stimulus categories.

## Results

### Basic characteristics

**Figure 1:**
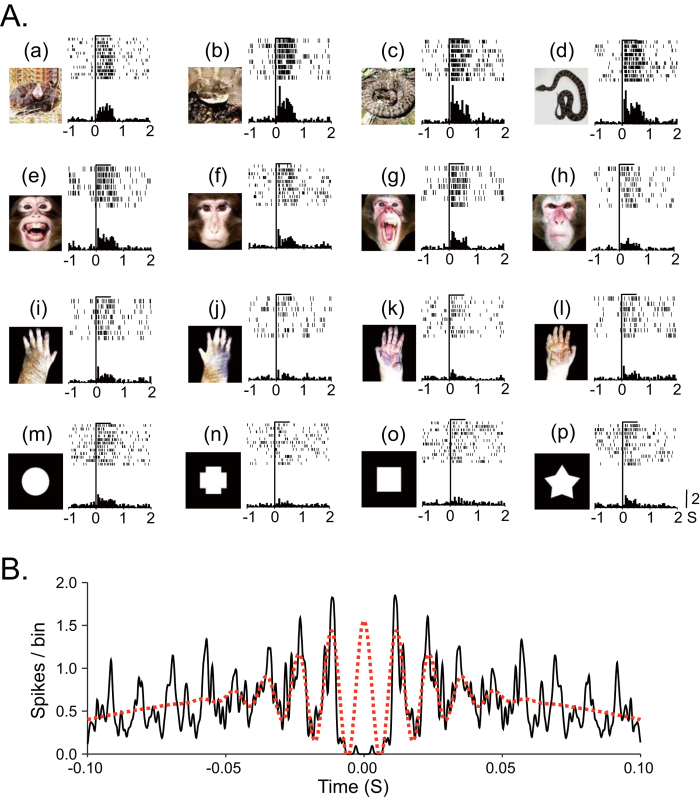
An example of a pulvinar neuron that responded most strongly to snakes. (**A, a–l**). Raster displays of neuronal activities and their summed histograms in response to each stimulus. (**a–d**) responses to snakes, (**e–h**) responses to monkey faces, (**i–l**) responses to monkey hands, and (**j–l**) responses to simple geometrical shapes. Horizontal bars above the raster displays indicate the stimulus presentation periods (500 ms). Vertical line in each of the raster displays and histograms indicates the stimulus onset. Calibration at the right bottom of the figure indicates the number of spikes per trial in each bin. Bin width = 50 ms. (**B**) An autocorrelogram of this neurons in the Early phase. Bin width = 0.1 ms. Ordinate indicates spikes/bin. Red dotted line indicates a wave of significant gamma oscillation. Illustrations are original drawings by Q. V. Le and H. Nishijo; snakes were photographed by Mr. I. Hoshino and Mr. D. Hillman.

Of 745 neurons recorded, 115 neurons responded to visual stimuli (responsive neurons). Of these 115 responsive neurons, 91 neurons were tested with all stimuli and were used to analyze gamma oscillations. Figure 1A shows an example of a pulvinar neuron that responded strongly to snakes. This neuron responded strongly to all four snake images (Fig. 1A, a–d) and less to other stimuli (e–p). Gamma oscillations of pulvinar neuronal activity were analyzed in three phases around the stimulus onset (Pre-stimulus: 500 ms before stimulus onset; Early: 0–200 ms after stimulus onset; and Late: 300–500 ms after stimulus onset). Figure 1B shows an example of gamma oscillation in Early phase during presentation of snakes. Of 91 responsive neurons, 79 neurons showed significant gamma oscillation for at least one of the four categories of the stimuli in one or more phases. These neurons were located in the medial and dorsolateral pulvinar^23^. Table 1 shows the number of pulvinar neurons with significant gamma oscillation activity during presentation of each category of the stimuli in the three phases.

### Comparison of gamma oscillation among the 4 stimulus categories

**Figure 2:**
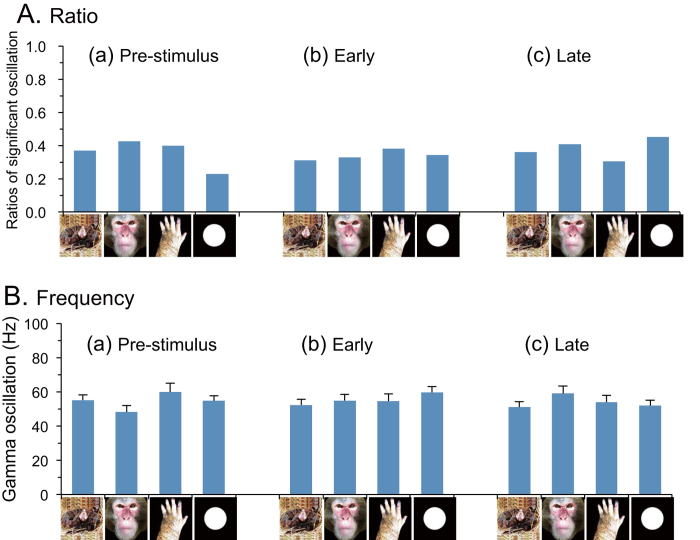
(**A**) Comparison of ratios of gamma oscillating neurons (number of gamma oscillating neurons/number of responsive neurons) among the three phases around stimulus onset. There was no significant difference in the ratios of gamma oscillating neurons among 3 phases nor among four categories of the stimuli. (**B**) Comparison of mean frequency of gamma oscillation among the three phases around stimulus onset. There was no significant difference in the frequency of gamma oscillation among 3 phases nor among four categories of the stimuli. Illustrations are original drawings by Q. V. Le and H. Nishijo; a snake was photographed by Mr. D. Hillman.

Figure 2A shows ratios of gamma oscillating neurons to the total of the responsive neurons (n = 91) in the three phases. The ratios among the four categories ranged from 20 to 50% but these differences were not statistically significant (χ2 tests, p > 0.05). Furthermore, there were no significant differences in ratios of gamma oscillating neurons among the 3 phases in each of the four categories (χ2 tests, p > 0.05). Figure 2B shows the comparison of mean frequencies of gamma oscillation. The pulvinar neurons showed 50–70 Hz gamma oscillation. However, a statistical analysis by two-way ANOVA indicated that there were no significant main effects of stimulus category [F(3, 192) = 0.554, p > 0.05] and phase [F(2, 192) = 0.140, p > 0.05], nor significant interaction between stimulus category and phase [F(6, 192) = 1.312, p > 0.05].

**Figure 3:**
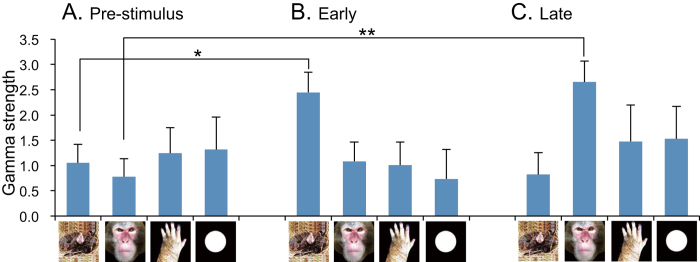
Comparison of gamma strength among the three phases around stimulus onset. *p < 0.05; **p < 0.01. Illustration is original drawing by Q. V. Le and H. Nishijo; a snake was photographed by Mr. D. Hillman.

**Figure 4:**
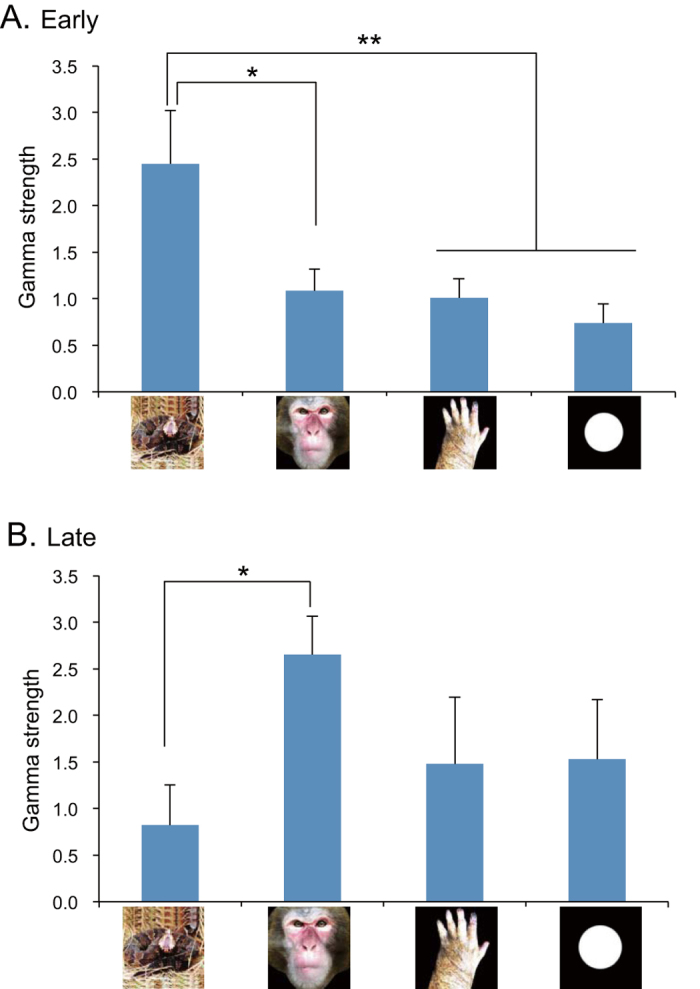
Comparison of gamma strength among the four categories of the stimuli in Early phase (A) and Late phase (B). *p < 0.05; **p < 0.01. Illustrations are original drawings by Q. V. Le and H. Nishijo; a snake were photographed by Mr. D. Hillman.

Figure 3 shows the comparison of mean strength of gamma oscillation (gamma strength). A statistical analysis by two-way ANOVA indicated that there were no significant main effects of stimulus category [F(3, 107) = 0.271, p > 0.05] and phase [F(2, 107) = 0.986, p > 0.05]. Nevertheless, there was a significant interaction between stimulus category and phase [F(6, 107) = 3.187, p < 0.05]. Post-hoc multiple comparisons indicated that mean gamma strength for snakes was significantly greater in Early phase than in Pre-stimulus phase (Bonferroni test, p < 0.05), while mean gamma strength for monkey faces was significantly greater in Late phase than in Pre-stimulus phase (Bonferroni test, p < 0.05). Figure 4A indicates subsidiary comparison of gamma strength among 4 categories in Early phase by one-way ANOVA. The statistical results indicated a significant main effect [F(3, 39) = 3.883, p < 0.016]. Post-hoc multiple comparisons indicated that mean gamma strength was significantly greater for snakes than for monkey faces (Tukey test, p < 0.05), monkey hands (Tukey test, p = 0.057), and simple geometrical patterns (Tukey test, p = 0.053). Figure 4B indicates subsidiary comparison of gamma strength among 4 categories in Late phase by one-way ANOVA. The statistical results indicated a significant main effect [F(3, 28) = 3.188, p < 0.027]. Post-hoc multiple comparisons indicated that mean gamma strength was significantly greater for monkey faces than for snakes in Late phase (Tukey test, p < 0.05).

To analyze these characteristics in detail, gamma oscillations (30–80 Hz) in the 200-ms period during 150–350 ms after stimulus onset (Mid-phase) were similarly analyzed. The results indicated that there were no significant differences in ratios of gamma oscillating neurons, among the four categories of the stimuli in Mid-phase (Supplementary Results). These results indicated that these characteristic changes were specific to Early and Late phases. Second, since gamma oscillation includes wide range of frequencies (i.e., 30–80 Hz), the gamma band was divided into two frequency bands; low gamma (30–50 Hz) and gamma (50–80 Hz). Separate analyses of oscillations in the two gamma bands indicated that both low and high gamma bands showed similar trends to those in full gamma band (Supplementary Results).

## Discussion

This study demonstrated that individual monkey pulvinar neurons showed gamma oscillation during visual discrimination. Although previous studies reported pulvinar involvement in gamma oscillation^36^,^37^, the present study provides the first evidence that activity of individual pulvinar neurons oscillates in gamma band frequencies. In the forebrain structures, parvalbumin-positive interneurons are specifically implicated in the generation of gamma oscillation in rodents^38^,^39^,^40^, and the pulvinar includes parvalbumin-positive neurons as local circuit neurons in cats^41^,^42^. Gamma oscillations have been reported in the SC^43^,^44^, which sends visual information to the pulvinar^45^,^46^. These neural circuits may contribute to gamma oscillation in the pulvinar.

As predicted, we found that images of snakes elicited strong gamma oscillations earlier than did other stimuli. Since low-level features of the snake photos were different from those of other categories (see Methods), this characteristic might be ascribed to these differences rather than to snakes themselves. However, it is unlikely. Our previous study indicated that pulvinar neuronal responses were markedly attenuated by scrambling the photos^23^. We thus further tested four pulvinar neurons with significant gamma oscillation in response to snakes with scrambled photos of the snakes. The results indicated that gamma band oscillation disappeared in response to the scrambled snakes in all four neurons. These results strongly suggest that features of snakes are important to induce gamma oscillation. Growing evidence indicates that the SC and pulvinar function as a coarse and quick visual processing module to detect threating stimuli, and gamma oscillation might facilitate perceptual processing by feedforward processes. Our findings suggest that images of snakes elicit gamma oscillation in the subcortical visual system, including the pulvinar, via fast bottom-up information processing that then activates the cortical visual system to hold attention. Consistent with this idea are findings that inactivation of the pulvinar decreased gamma oscillation in the visual cortex^36^,^37^ and salience of the stimulus^47^. Furthermore, it has been reported that the strength of visual stimulus-induced gamma oscillations in the visual cortex predicted the speed with which subjects detected stimulus changes^48^, and gamma power was associated with conscious recognition of visual stimuli^49^. These findings suggest a functional role of gamma oscillation in efficient visual processing, and the present results suggest that snakes could be detected efficiently by gamma oscillation.

We previously reported that macaque pulvinar neurons differentially respond to facial stimuli^21^. In that study, over 50% of face responsive neurons responded 200 ms after stimulus onset. In the present study, gamma strength for monkey faces was greater in the Late phase, later than with snakes. Previous studies have defined two temporal types of gamma oscillations; ‘early gamma’ before 150 ms after stimulus onset, and ‘late gamma’ later than 200 ms after stimulus onset. Early gamma might be related to bottom-up processes, while late gamma might be related to top-down processes to interpret and utilize the information resulting from the processes by the early gamma (reviewed by Herrmann *et al.* 2004)^50^. Faces are complex social stimuli for primates, suggesting that faces might activate sophisticated memories that are stored in the distributed cortical areas. Consistent with this idea, gamma oscillation is reported to be involved also in higher cognitive processes such as memory retrieval^51^. The pulvinar has intimate and reciprocal connections with various cortical association areas^52^, which are also directly connected to each other^53^,^54^. Furthermore, inactivation of the pulvinar reduced information transfer between the visual association areas^32^. These findings suggest that gamma oscillation in the Late phase might be involved in these processes interrelating the association cortices.

The primate visual system has been argued to have evolved under the evolutionary pressure of snakes that would have given an advantage to individuals that could react quickly to snakes^2^,^3^. Consistent with this hypothesis, as described above, several behavioral studies have shown that humans and monkeys respond faster to snakes than other stimuli. The present results showed stronger gamma oscillation of pulvinar neurons in different periods in response to snake (0–200 ms) and face images (300–500 ms). An early increase in gamma oscillation elicited by snake images might contribute to rapid snake detection by feedforward processing, whereas a later increase in gamma strength might reflect more sophisticated social information processing in complex cortico-pulvinar-cortical pathways. The present results provide electrophysiological evidence that gamma oscillation can occur at individual neurons in the pulvinar, and highlight distinct visual information processing of snakes and faces in gamma oscillation.

## Methods

### Subjects

Two adult (one female and one male) macaque monkeys (*Macaca fuscata*) weighing 7.2–9.5 kg were used. The monkeys were deprived of water in their home cage and received juice as a reward during training and recording sessions. Supplemental water and vegetables were given after each day’s session. To assess the monkeys’ health, their weight was routinely monitored. The monkeys were treated in strict compliance with the United States Public Health Service Policy on Human Care and Use of Laboratory Animals, the National Institutes of Health Guide for the Care and Use of Laboratory Animals, and the Guidelines for the Care and Use of Laboratory Animals of the University of Toyama. This study was approved by the Committee for Animal Experiments and Ethics at the University of Toyama.

### Experimental setup

The monkey sat in a monkey chair 68 cm away from the center of a 19-inch computer display for behavioral tasks during the training and recording sessions in a shielded room. The CRT monitor was set so that its center was on the same horizontal plane as the monkey’s eyes. The monkey chair was equipped with a responding button, which was positioned so that the monkey could easily manipulate it. An infrared charge-coupled device (CCD) camera for eye-movement monitoring was firmly attached to the chair by a steel rod. During training and recording sessions, the monkey’s eye position was monitored with 33 ms time resolution by an eye-monitoring system^55^. The juice reward was accessible to the monkey through a small spout controlled by an electromagnetic valve. A visual stimulus generator (ViSaGe MKII Visual Stimulus Generator, Cambridge Research Systems, UK) controlled the electromagnetic valve, the timing of visual stimuli onset.

### Visual stimuli

**Figure 5:**
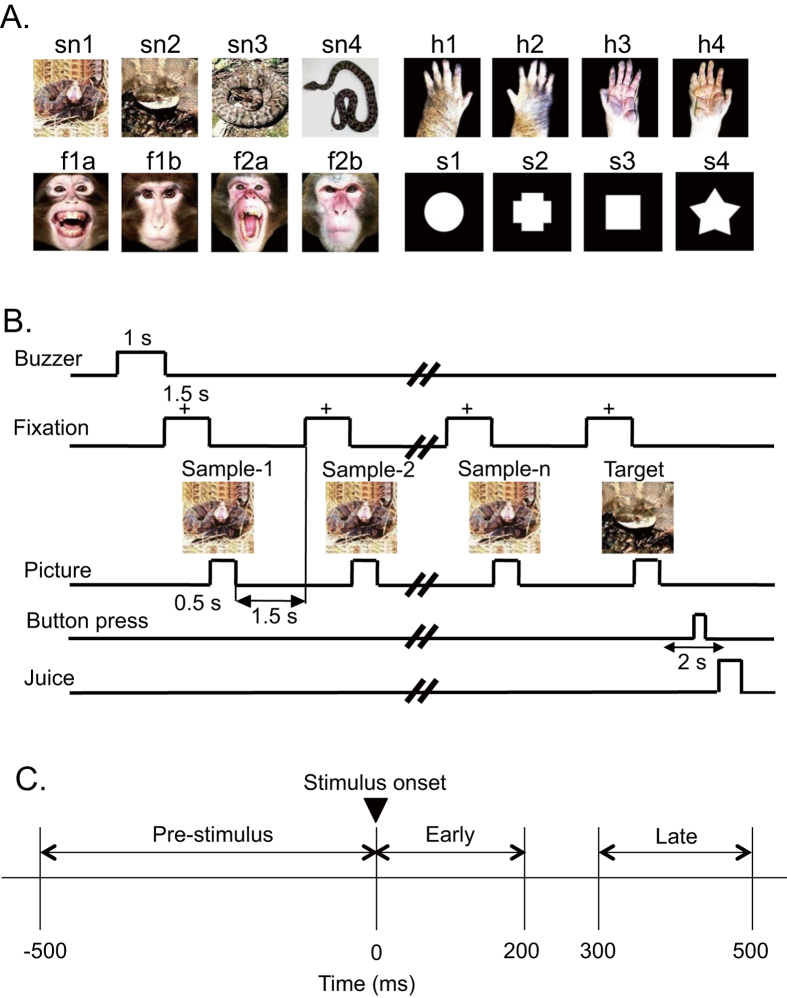
Visual stimuli (A) and delayed nonmatching-to-sample (DMNS) task (B) used in the present study, and three task phases for an analysis (C). (**A**) Sixteen photos of four categories of the stimuli including snakes, monkey faces, monkey hands, and simple geometrical patterns. (**B**) Stimulus sequence in the DMNS task in which stimuli were sequentially presented with a delay. (**C**) Three phases during which gamma oscillation was analyzed. Illustrations are original drawings by Q. V. Le and H. Nishijo; snakes were photographed by Mr. I. Hoshino and Mr. D. Hillman.

Figure 5A shows the stimulus set, consisting of photographs of snakes, monkey faces (neutral and expressive faces), and monkey hands, and drawings of simple geometrical patterns (circle, cross, square and star), used in the present study. The stimuli were 256 digitized RGB color-scale images with their resolution of 227 × 227 pixels. Stimuli were presented on a black background of 0.7 cd/m^2^ with their centers at the center of the display. The luminance of these color stimuli was almost identical (6.005–6.445 cd/m^2^) [luminous intensity (total luminance) ranged from 38.432 to 41.248 mcd]. Luminance of the white areas inside the simple geometric patterns was 36.5 cd/m^2^ (total luminance of the circle, cross, square, and star was 33.368, 32.676, 32.555, and 31.822 mcd, respectively). These stimuli were displayed on a CRT monitor with a resolution of 640 × 480 pixels, and the size of the stimulus area was 5–7 × 5–7°.

Furthermore, low-level features of the visual stimulus, i.e., contrast, color histograms, and spatial-frequency power distribution were calculated and compared across the four categories. The comparisons indicated that low-level features (color histogram, spatial-frequency power distribution) of snakes were significantly different from those of other categories (see Supplementary Methods).

### Behavioral tasks

The monkeys were trained to perform a sequential delayed nonmatching-to-sample (DNMS) task that required the discrimination of visual stimuli (Fig. 5B)^23^. The task was initiated by a buzzer tone. Then, a fixation cross appeared at the center of the display. When the monkeys fixated on the cross for 1.5 s within 0.5–1.0° window, a sample stimulus was presented for 500 ms (sample phase). Then, after an interval of 1.5 s, the same stimulus appeared again for 500 ms, and this occurred between one and four times (selected randomly for each trial). Finally, a new stimulus was presented (target phase). When the target appeared, the monkey was required to press the button within 2 s to receive a juice reward (0.8 mL). When the monkey failed to respond correctly during the target phase or to press the button before the target phase, the trials were aborted, and a 620-Hz buzzer tone was sounded. The intertrial intervals were 15–25 s.

### Electrophysiological procedures and data acquisition

The monkeys were trained to perform DNMS task for 3 h/day, 5 day/week. The monkeys reached a 96% correct-response rate after 3 months of training^23^,^27^. After completion of this training period, a head-restraining device, which was a U-shaped plate made of epoxy resin, was attached to the skull under aseptic conditions^23^,^27^. After the monkeys relearned the DNMS task and were correct at least 85% of the time, we commenced recording neuronal activity from each hemisphere in both subjects. A glass-insulated tungsten microelectrode (0.8–1.5 MΩ at 1 kHz) was stereotaxically inserted into the pulvinar vertically to the orbitomeatal plane. The analog signals of the neuronal activities, visual stimulus triggers, juice rewards, button presses, and X-Y eye position coordinates were digitized at a 40-kHz sampling rate and stored in a computer through a multichannel acquisition processor (Plexon Inc., Dallas, TX) system. The digitized neuronal activities were isolated into single units by their waveform components with the Offline Sorter program (Plexon Inc.). The data that were used in the present study were previously reported in Le *et al.* (2013, 2014)^23^,^27^, and more details of the procedures can be found in those studies.

### Analysis of the basic characteristics of pulvinar neurons

We analyzed the activity of single neurons during the 500-ms period after (post) the onset of stimulus presentation in the sample phase, but we did not analyze the activity of single neurons in the target phase. Only the stimuli that were presented more than five times in the sample phase across trials were analyzed. The baseline firing rate was defined as the mean firing rate during the 100-ms pre period. The significance of the excitatory or inhibitory responses to each stimulus was determined by comparing between the 100-ms pre and 500-ms post periods with a Wilcoxon signed-rank test. P values less than 0.05 were considered statistically significant (responsive neurons).

### Periodicity of spike firings

For each responsive neuron, periodic firing patterns in the 30–80 Hz range during the DNMS task were analyzed in the 3 phases for each stimulus category (Fig. 5C); 500-ms period before stimulus onset (Pre-stimulus phase), 200-ms period after stimulus onset (Early phase), and 200-ms period during 300–500 ms after stimulus onset (Late phase). An auto-correlogram over 200 ms (bin size 0.1 ms) was calculated in each phase and filtered with the Gaussian filter (full width at half maximum, 1 ms). Then, according to König (1994)^56^ and Engel *et al.* (1990)^56^, the primary oscillation frequency between 10 and 150 Hz was calculated by non-linear fitting of the following function to the auto-correlogram.


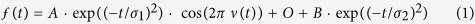


Where the first term represents Gabor function; the second term (*O*) is an offset; the third term represents a Gaussian function to consider a central modulation of the auto-correlogram; *t* is time; *A*, *σ*_*1*_ and *ν* are amplitude, decay constant, and wave frequency of the Gabor function, respectively; and *B* and *σ*_*2*_ are amplitude and width of the Gaussian function, respectively. Frequency of oscillation of a given neuron corresponds to wave frequency of the Gabor function (*ν*). Although the same function and the algorithm for non-linear regression were used as reported by König (1994)^56^, the criteria were slightly modified following Engel *et al.* (1990)^57^ and Matsumoto *et al.* (2012)^58^. A given neuron was considered to be significantly oscillated in a given frequency (*ν*) and in a given phase according to the following three criteria: (1) the function was regressed with the effective coefficient of the amplitude (*A*) and frequency (*ν*) (p < 0.05); (2) the decay constant (*σ*_*1*_) was larger than 1/ ν*0.8, which means that the fitted function had at least one satellite peak^57^,^58^; and (3) the number of spikes within the auto-correlogram was >50.

If a neuron showed the amplitude (*A*) and offset (*O*) to be simultaneously statistically significant, we calculated the strength of oscillation by the ratio between *A* and *O* for that neuron.

### Statistical analysis of gamma oscillation

The percentages of gamma oscillating neurons, frequency and strength of oscillation in response to each stimulus category (snakes, monkey faces, monkey hands, and simple geometrical patterns) in each phase (Pre-stimulus, Early, and Late) were calculated. The ratios of gamma oscillating neurons were compared with Chi-square tests. Frequency and strength of oscillation among the 3 phases were compared using two-way analysis of variance tests (ANOVA) with post hoc tests using Bonferroni correction. All statistical analyses were performed using the SPSS software package (ver. 19, IBM Corporation, Armonk, NY, USA). Any differences were considered statistically significant with p < 0.05.

## Additional Information

**How to cite this article**: Van Le, Q. *et al.* Snakes elicit earlier, and monkey faces, later, gamma oscillations in macaque pulvinar neurons. *Sci. Rep.* **6**, 20595; doi: 10.1038/srep20595 (2016).

## Supplementary Material

Supplementary Information

## Figures and Tables

**Table 1:** Number of pulvinar neurons with significant gamma oscillation activity during presentation of each category of the stimuli.

	Pre-stimulus	Early	Late
Snakes	23	18	23
Monkey faces	23	20	22
Monkey hands	14	16	11
Simple patterns	8	12	14
