# Supplementary material for: Snakes elicit earlier, and monkey faces, later, gamma oscillations in macaque pulvinar neurons
Source: Sci Rep. 2016 Feb 8;6:20595. doi: 10.1038/srep20595 (PMC4744932; doi:10.1038/srep20595)
Supplement: Supplementary Information [file srep20595-s1.pdf]

Snakes elicit earlier, and monkey faces, later, gamma oscillations in macaque pulvinar neurons

by

Quan Van Le[1,4], Lynne A. Isbell[2], Jumpei Matsumoto[1], Van Quang Le[1], Hiroshi Nishimaru[1], Etsuro Hori[1], Rafael S. Maior[3,5], Carlos Tomaz[3], Taketoshi Ono[1] and Hisao Nishijo[1]

Supplementary Information

Supplementary Results

To analyze these characteristics in detail, the gamma oscillation (30-80 Hz) in the 200-ms period during 150-350 ms after stimulus onset (Mid-phase) were analyzed as in Early and Late phases. The results indicated that there were no significant differences in ratios of gamma oscillating neurons among the four categories of the stimuli in Mid-phase (Supplementary Fig. S1). These characteristic changes were specific to Early and Late phases. Second, since gamma oscillation includes a wide range of frequencies (i.e., 30-80 Hz), the gamma band was divided into two frequency bands; low gamma (30-50 Hz) and gamma (50-80 Hz). In low gamma band (30-50 Hz), there was no significant difference in ratio of low gamma oscillating neurons, nor mean low gamma frequencies (Supplementary Fig. S2), consistent with the results in full gamma band (30-80 Hz). However, there was no significant difference in mean low gamma strength although a similar trend to that in full gamma band was observed (Supplementary Fig. S2). These insignificant results are ascribed to the smaller number of low gamma oscillating neurons as a consequence of narrowing the range of frequency. In high gamma band (50-80 Hz), there was no significant difference in ratio of high gamma oscillating neurons, nor mean high gamma frequencies (Supplementary Fig. S3), consistent with the results of full gamma band (30-80 Hz). However, there were significant differences in high gamma strength: mean high gamma strength tended to be greater for snakes than for monkey faces in Early phase (Tukey test, $p < 0.05$), while it was significantly greater for monkey faces in Late phase than Pre-stimulus phase (Bonferroni test, $p < 0.05$). These results indicated that the results in both low and high gamma bands showed similar trends to those in full gamma band.

Supplementary Methods

Low-level features of the visual stimuli

Low-level features of the visual stimuli (contrast, color histograms, and spatial-frequency power distribution) were calculated and compared across the four categories, as follows. Michelson contrast of each stimulus was calculated and compared among the categories by

one-way ANOVA and post-hoc Bonferroni tests. Michelson contrast was not significantly different among the four categories [one-way ANOVA, $F(3, 14) = 0.622$, $p = 0.612$].

A color histogram consisting of four equally spaced bins was calculated for each of red, green, and blue channels of each stimulus. For each of the color channel, the histogram was compared among the four categories by two-way ANOVA (bins x categories) and post-hoc simple main effect analyses. These comparisons revealed significant interaction between category and bin [red: $F(9, 56) = 20.62$, $p = 9.0 \times 10^{-15}$; green: $F(9, 56) = 16.54$, $p = 7.6 \times 10^{-13}$; blue: $F(9, 56) = 9.34$, $p = 1.7 \times 10^{-8}$]. Overall, in all of the channels, there was a tendency that the snake-category contained more middle-intensity pixels and fewer low-intensity pixels than other categories (simple main effect test, $p < 0.05$), which might be ascribed to the difference in their backgrounds, i.e., natural backgrounds vs. black-painted backgrounds.

Two-dimensional power spectrum of each visual stimulus was calculated through 2-D fast Fourier transform with a MATLAB function (fft2). Then, the spectrum was reduced to a one-dimensional function of spatial frequency by averaging powers over orientations [1]. Total power of low and high spatial frequency components were calculated as total powers in the 1-D function from 1 to 8 cycle/image and from 9 to 113 cycles/image, respectively. Each of the low and high spatial frequency powers was compared among categories by one-way ANOVA and post-hoc Bonferroni tests. These comparisons indicated that the powers of low spatial frequency components were significantly smaller in the snakes (82.2 ± 0.4 dB) than the simple-patterns (84.1 ± 0.8 dB) [$F(3, 14) = 4.02$, $p = 0.030$, one-way ANOVA; $p = 0.038$, Bonferroni tests]. On the other hand, the powers of high spatial frequency components were significantly larger in snakes (74.1 ± 1.2 dB) than faces (68.1 ± 0.8 dB) and simple-patterns (66.4 ± 1.1 dB) [$F(3, 14) = 11.7$, $p = 4.3 \times 10^{-4}$, one-way ANOVA; $p < 0.05$, Bonferroni tests].

These comparisons indicated that low-level features (i.e., color histogram, spatial-frequency power distribution) of snakes were significantly different from those of other categories.

References

1. Simoncelli, E. P. & Olshausen, B. A. Natural image statistics and neural representation. *Ann. Rev. Neurosci.* **24**, 1193-1216 (2001).

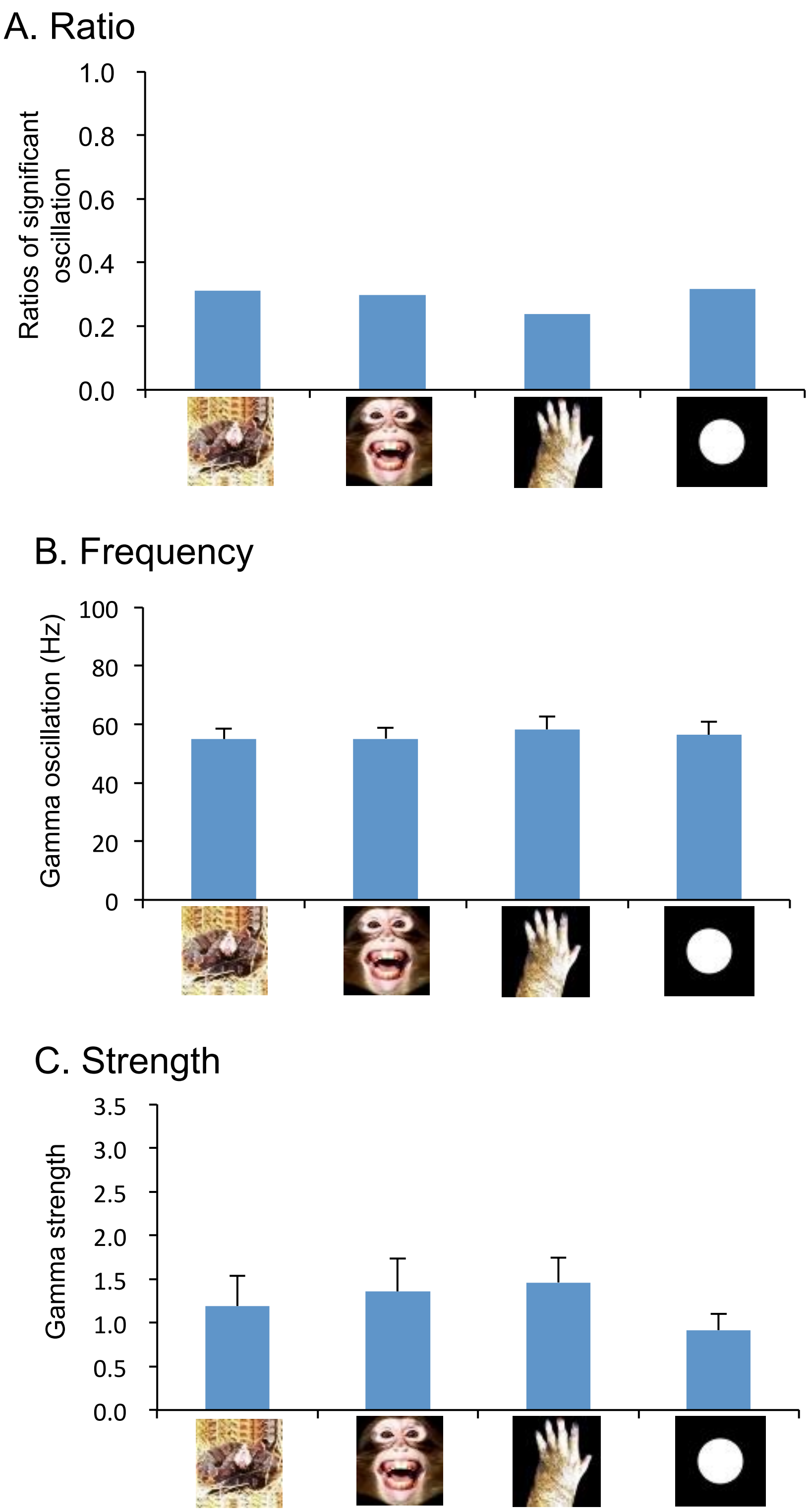

Supplementary Fig. S1. (A) Comparison of ratios of gamma oscillating neurons among the four categories of the stimuli in Mid phase. There was no significant difference in the ratios of gamma oscillating neurons among 3 phases nor among four categories of the stimuli ($\chi 2$ tests, $p>0.05$). (B) Comparison of mean frequency of gamma oscillation among the four categories of the stimuli in the mid phase. There was no significant difference in the frequency of gamma oscillation among four categories of the stimuli [$F(3,63) = 0.122$, $p = 0.947$]. (C) Comparison of mean gamma strength among the four categories of the stimuli in Mid phase. There was no significant difference in mean strength among four categories of the stimuli [$F(3,33) = 0.324$, $p = 0.808$].

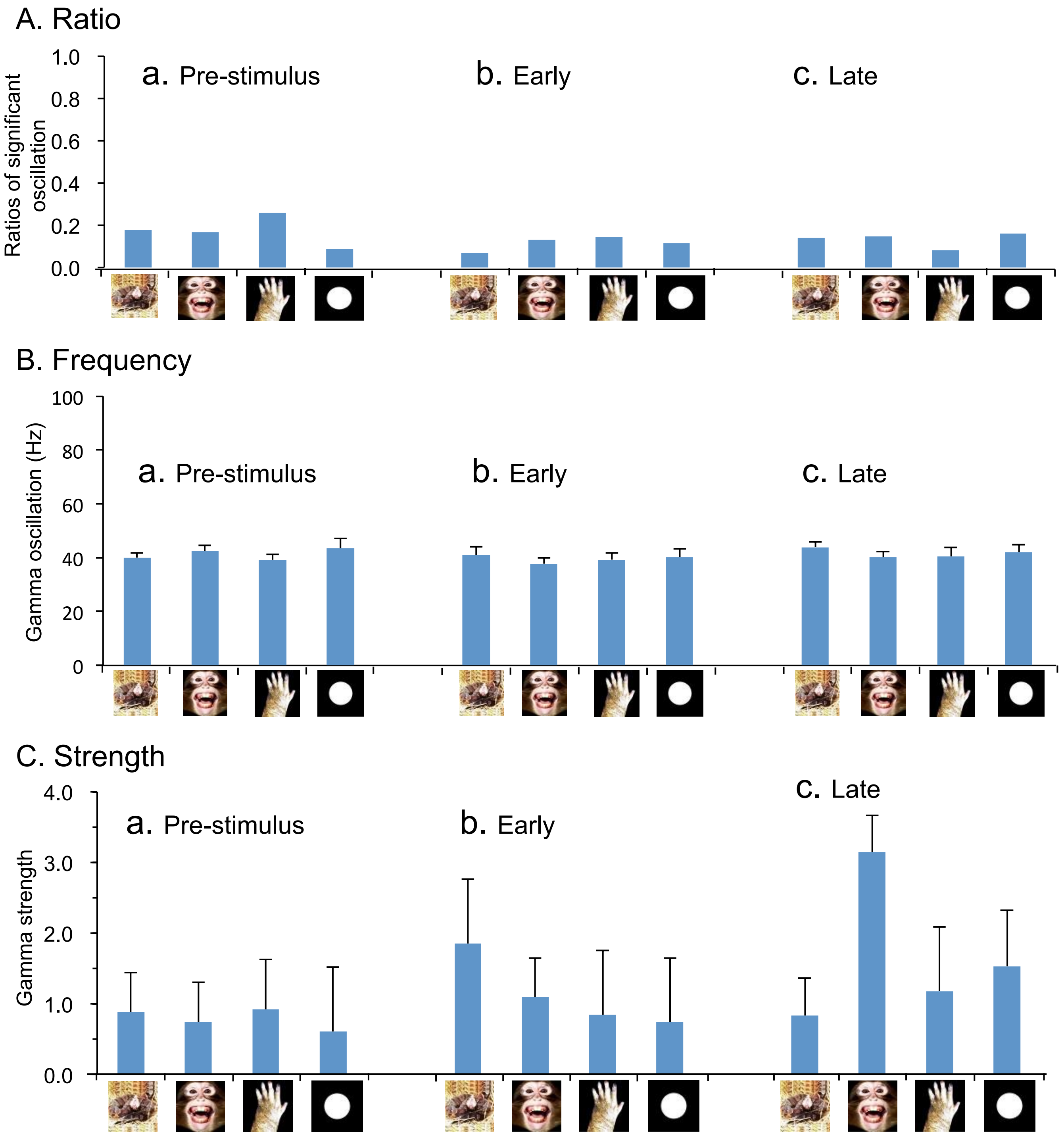

Supplementary Fig. S2. (A) Comparison of ratios of low gamma oscillating neurons among the three phases around stimulus onset. There was no significant difference in the ratios of low gamma oscillating neurons among 3 phases nor among four categories of the stimuli (χ2 tests, p>0.05). (B) Comparison of mean frequency of low gamma oscillation among the four categories of the stimuli and among the three phases. There was no significant difference in mean frequencies among 3 phases [$F(2,67) = 0.643$, $p = 0.529$], nor among the four categories of the stimuli [$F(3,67) = 0.549$, $p = 0.651$]. (C) Comparison of low gamma strength among the four categories of the stimuli and among the three phases. There was no significant difference in the ratios of gamma oscillating neurons among 3 phases [$F(2,54) = 1.597$, $p = 0.212$], nor among the four categories of the stimuli [$F(3,54) = 0.752$, $p = 0.526$].

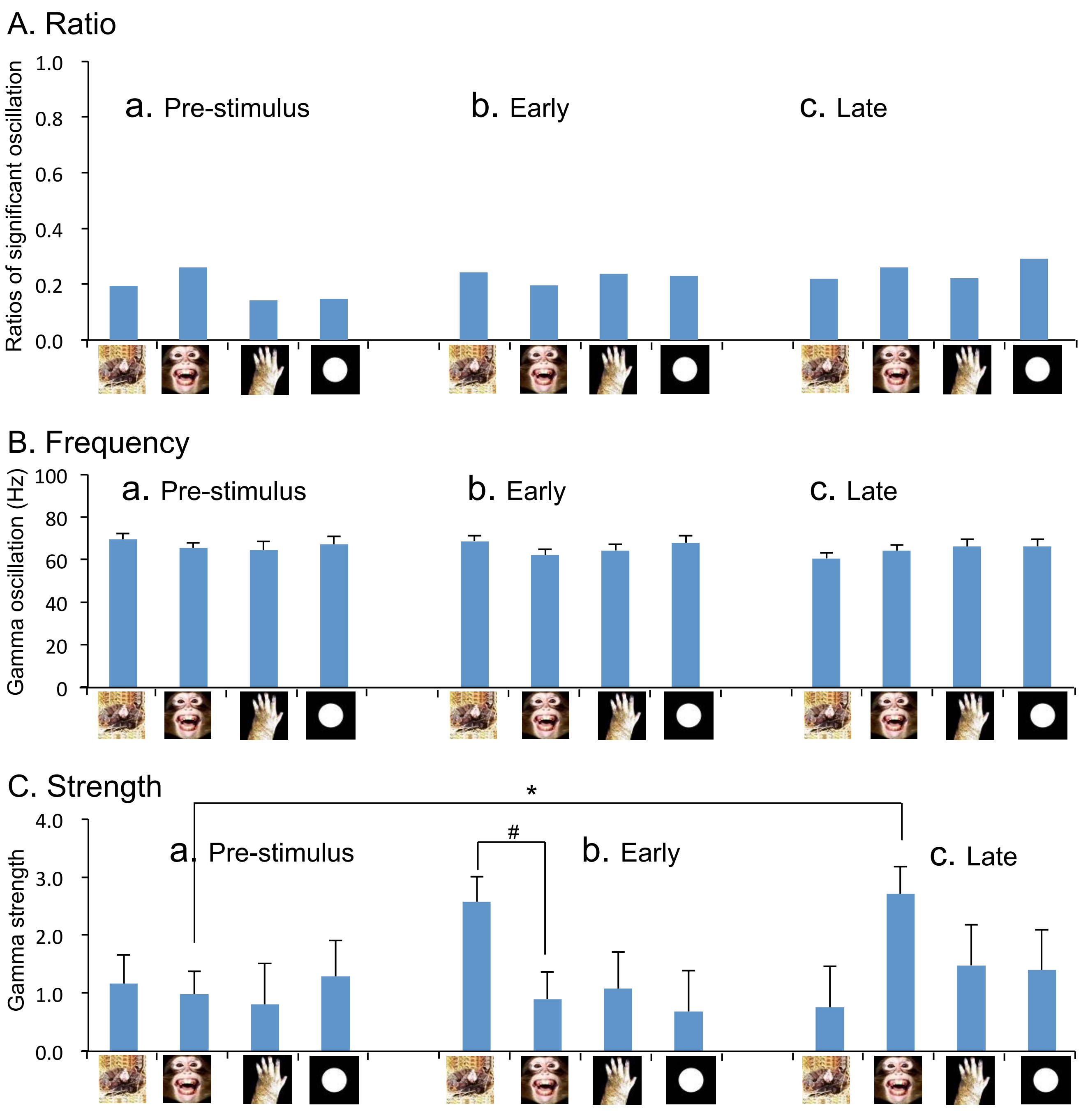

Supplementary Fig. S3. (A) Comparison of ratios of high gamma oscillating neurons among the three phases around stimulus onset. There was no significant difference in the ratios of high gamma oscillating neurons among 3 phases, nor among four categories of the stimuli ($\chi 2$ tests, $p>0.05$). (B) Comparison of mean frequency of high gamma oscillation among the four categories of the stimuli and among the three phases. There was no significant difference in mean frequencies among 3 phases [$F(2,114) = 0.559$, $p = 0.573$], nor among four categories of the stimuli [$F(3,114) = 0.669$, $p = 0.573$]. (C) Comparison of high gamma strength among the four categories of the stimuli and among the three phases. There was a significant interaction between phase and category [$F(6,67) = 2.243$, $p = 0.049$]. *, $p < 0.05$ (Bonferroni test); #, $p < 0.1$ (Tukey test after subsidiary one-way ANOVA).
